# Non-Coding RNAs in Pediatric Airway Diseases

**DOI:** 10.3390/genes8120348

**Published:** 2017-11-27

**Authors:** Beata Narożna, Wojciech Langwiński, Aleksandra Szczepankiewicz

**Affiliations:** Laboratory of Molecular and Cell Biology, Department of Pediatric Pulmonology, Allergy and Clinical Immunology, Poznan University of Medical Sciences, 60-512 Poznan, Poland; b.narozna@gmail.com (B.N.); wlangwinski654@gmail.com (W.L.)

**Keywords:** miRNA, long non-coding RNA, asthma, cystic fibrosis, viral infection, biomarkers

## Abstract

Non-coding RNAs (ncRNAs) are involved in the regulation of numerous biological processes and pathways and therefore have been extensively studied in human diseases. Previous reports have shown that non-coding RNAs play a crucial role in the pathogenesis and aberrant regulation of respiratory diseases. The altered expression of microRNAs (miRNAs) and long non-coding RNAs in blood and also locally in sputum or exhaled breath condensate influences lung function, immune response, and disease phenotype and may be used for the development of biomarkers specific for airway disease. In this review, we provide an overview of the recent works studying the non-coding RNAs in airway diseases, with a particular focus on chronic respiratory diseases of childhood. We have chosen the most common chronic respiratory condition—asthma—and the most severe, chronic disease of the airways—cystic fibrosis. Study of the altered expression of non-coding RNAs in these diseases may be key to better understanding their pathogenesis and improving diagnosis, while also holding promise for the development of therapeutic strategies using the regulatory potential of non-coding RNAs.

## 1. Introduction

Non-coding RNAs (ncRNAs) are master regulators of gene expression that influence numerous biological pathways including proliferation, differentiation, and apoptosis, as well as immune response (innate and adaptive immunity, differentiation of T and B cells, inflammation, and tissue repair and remodeling (reviewed by [1])). Non-coding RNAs are grouped into housekeeping RNAs and regulatory RNAs. Housekeeping RNAs include those involved in ribosomal machinery, i.e. rRNAs, transfer RNAs (tRNAs), small nuclear RNAs (snRNAs) and small nucleolar RNAs (snoRNAs), whereas regulatory RNAs include short non-coding RNAs (less than 200 nucleotides) and long non-coding RNAs (longer than 200 nucleotides) [2,3]. The most commonly RNAs studied in respiratory diseases are short ncRNAs (mainly microRNAs, but recently also piwi-interacting RNAs (piRNAs) and long ncRNAs.

MicroRNAs (miRNAs) are small non-coding RNAs (18–25 nucleotides long) that regulate expression of target genes by inhibiting their translation via RNA interference [4]. They are synthesized as precursors in the nucleus, where they undergo maturation with several enzymatic reactions and are translocated to the cytoplasm where they exert their biological function in a protein complex. It has been predicted that, in humans, about 60% of mRNAs are targets for miRNA [5] and one miRNA may target more than 100 mRNAs [6]. Another group of small RNAs are piRNAs that play an important role in germline cells. They are involved mainly in transposon silencing and epigenetic DNA and histone modification in germinal cells and early embryos [7]. Recently, piRNAs have also been found in differentiated cells [8].

Long non-coding RNAs are heterogeneous group of non-coding RNAs with regard to origin and mechanism of action/function. Another interesting feature is that they may be expressed in both sense and antisense orientation relative to protein-coding genes [9]. Studies demonstrated that they are regulators of different cellular processes including chromatin structure changes, transcription and post-transcriptional processing, and intracellular trafficking [10]. Although their role in diseases has not yet been well characterized, it is known that they are involved in cancer development for example [11,12].

The most widely studied non-coding RNAs in airway diseases are miRNAs, but a few studies have also analyzed the role of other types (such as lncRNAs, piRNAs). The miRNAs were first studied as potential biomarkers in lung cancer (as reviewed by [13]). It was found that the differences in miRNAs with respect to normal and cancer lung tissue may be related to diagnosis and prognosis of the disease, while also offering novel therapeutic targets for lung cancer therapy [14]. In the last decade, dynamic progress has been made in discovering the role of ncRNAs in the pathogenesis and diagnosis of airway diseases.

The aim of this review is to provide an overview of the most current knowledge and to summarize existing data on ncRNAs as the causal factors involved in the pathogenesis of chronic airway diseases, as well as biomarkers of disease state. This review will focus on the two most common chronic childhood diseases of the airways, which have different genetics backgrounds: polygenic asthma, and monogenic cystic fibrosis, with particular emphasis on the central role of airway epithelium in the pathogenesis of these diseases and responses to environmental stimuli.

## 2. NcRNA Studies in Asthma

The development of microarrays and new-generation sequencing techniques in the past few decades has created the possibility of determining the expression and sequencing of many non-coding RNAs in a single reaction. This progress has significantly increased our knowledge about the role of non-coding RNAs in the pathogenesis of allergic asthma [15,16,17]. Numerous papers have highlighted the potential contribution of non-coding RNAs in the pathogenesis of allergic asthma, and miRNAs seem to be a focus in these studies. A summary of studies of ncRNA in asthma is shown in Table 1.

Several studies on miRNA expression profiling report the importance of miRNAs in the regulation of airway inflammation in asthma. In one of the first studies, Liu et. al. demonstrated that 83 miRNA genes were differentially expressed (36 up-regulated and 47 down-regulated) in the blood of asthmatic children as compared to control subjects [18]. Using a murine model of asthma, the authors demonstrated that miR-221 exhibited increased expression in the lungs of sensitized animals, similar to that observed in the blood of asthmatic children [18]. MiR-221 in silico was predicted to target the *Spred-2* gene, encoding Sprouty-related protein with EVH1 domain-2, that was downregulated in the lungs of sensitized mice. Elevated expression of miR-221, that correlated with increased eosinophil counts in bronchoalveolar lavage fluid (BALF), was also reported in the lungs of allergic animals [19]. Moreover, inhibition of miR-221 with a synthetic inhibitor significantly reduced eosinophil numbers in BALF from asthmatic mice [20], whereas its overexpression stimulated P815 mast cells to increase synthesis and secretion of IL-4, crucial in Th2 polarization during allergic inflammation [19]. Apart from cytokine production, miR-221 overexpression also enhanced migration, adhesion, and antigen-induced mast cell degranulation in mouse bone marrow-derived mast cells (BMMCs) [21]. This miRNA was also suggested to be involved in airway smooth muscle proliferation, leading to thickening of the bronchial wall, airflow limitation, and dyspnea [22].

A recent study on miRNA profiling in childhood asthma showed that several miRNA genes, including let-7e, miR-98, and miR-497 demonstrated an over two-fold up-regulation in moderate-to-severe asthma as compared to the control group [46]. Moreover, in the same study the expression of HS_101, HS_206, miR-208, miR-518f, and miR-606 were increased in patients with allergic asthma as compared to non-allergic subjects. A previous functional study of the let-7 family found that let-7 inhibition suppressed production of allergic cytokines, suggesting its pro-inflammatory function.

Another miRNA widely studied in bronchial asthma is miR-21, which showed elevated levels in the serum of asthmatic patients and was suggested as a potential biomarker for allergic asthma [26]. Lu et. al., using transgenic mice with IL-13 overexpression, found 21 miRNAs differentially expressed in animals with induced inflammation as compared to transgenic mice that did not undergo induced allergic inflammation [27]. All these miRNAs were associated with the IL-13 pathway, crucial during Th2 polarization. Of these, miR-21 demonstrated the highest expression in sensitized mice when compared to control animals. The same miRNA also showed increased expression in mice sensitized to ovalbumin (OVA) or *Aspergillus fumigatus,* suggesting its essential and universal role in the allergic sensitization, regardless of the allergen [27]. Further studies, performed by the same authors, confirmed that knock-out of the miR-21 gene significantly reduced Th2-dependent inflammation in mice exposed to OVA [28]. Both miR-21 and miR-126 were overexpressed in OVA-sensitized mice [30], as well as in bronchial epithelial cells from asthmatic patients [29]. MiR-126 inhibition significantly decreased Th2-dependent response, namely allergen-induced hyper-responsiveness and eosinophil recruitment in sensitized animals [30,31].

Frequently studied in atopic asthma were also miR-146a and miR-146b, considered as transcription factor NF-kB-dependent, negative regulators of inflammatory genes [47]. It was shown that lipopolysaccharides (LPS) and pro-inflammatory cytokines (IL-1β) stimulated miR-146 synthesis, whereas genes involved in the Toll-like receptors pathway, *TRAF6* and *IRAK1*, were suggested as miR-146 target genes [32]. Studies performed on clinical samples confirmed elevated expression of miR-146a in the plasma of asthmatic children. Its expression may be also affected by variation in the miR-146 gene sequence. It was demonstrated by Jazdzewski et al. that a polymorphism (rs2910164) in pre-miR-146a significantly decreased the level of functional miRNA-146a [48] and this variant was associated with asthma in the Mexican female population [49].

Another candidate miRNA in atopic asthma is miR-499. Toraih et al. [50] demonstrated that a polymorphism (rs3746444), resulting in A>G substitution in the seed region of this miRNA gene, was associated with asthma risk in the population of Egyptian children and adults. The same polymorphism showed a significant association with spirometry results (increased forced expiratory volume in 1 s, FEV_1_) in a population of Korean patients with AG/GG genotype in comparison to patients with AA genotype [51]. Moreover, other miRNA gene polymorphisms (rs11614913 in miR-196a2 and rs2910164 in miR-146a) were associated with eosinophilic phenotype of asthma and increased bronchial hyper-responsiveness.

MiRNAs from the 146 family (miR-146a and miR-146b) were downregulated in circulating CD4+ and CD8+ T cells from severely asthmatic patients, but not in those with non-severe asthma [33]. It was shown that miR-146a and miR-146b negatively regulate the immune response [47] and their reduced expression may activate T-cells. In addition to studies of T cells, a number of reports have examined peripheral blood mononuclear cells and have shown miR-192 downregulation in mild asthmatics [34] and miR-211 and miR-485-3p upregulation in pediatric patients with asthma [18,20]. Another study of miR-146b and also 21 other miRNAs reported an association with spirometry results in asthmatic children [52]. Moreover, the authors demonstrated that 4 and 8 miRNA genes correlated with forced expiratory volume in 1 s (FEV_1_) and forced vital capacity (FVC), respectively, suggesting the potential role of circulating miRNAs as biomarkers for lung function in asthma.

Although peripheral blood is the most commonly studied material, it does not reflect specific changes in the airways upon inflammation. Less frequently used, but more specific for local inflammation, are sputum, BALF, and exhaled breath condensate. MiRNA profiling in BALF extracellular vesicles (EVs) [53] revealed 85 miRNAs that were differentially expressed between control mice and mice with allergic inflammation, induced by exposure to house dust mite (HDM). Moreover, 54 of these altered miRNAs were common between both EVs and the lung tissue from allergic animals. Furthermore, HDM sensitization significantly increased extracellular vesicle secretion, suggesting their potential role in transcellular communication upon allergen sensitization. Another miRNA study on exosomes from the BALF of asthmatic patients demonstrated 24 differentially expressed miRNAs, suggesting let-7 and miR-200 families as potential asthma biomarkers [24].

Studies analyzing miRNA expression profile in the sputum from mild-to-moderate asthmatic patients demonstrated elevated expression of miR-223-3p, miR-629-3p, miR-142-3p, miR-338, and miR-145 [35,36]. Furthermore, a strong association was found between increased expression of three miRNAs (miR-223-3p, miR-629-3p, and miR-142-3p), and severe asthma; these miRNAs were also negatively associated with spirometry results [36]. Another study performed in sputum of asthmatic patients demonstrated a significant downregulation of miR-155 as compared to controls [37]. Moreover, the expression of this miRNA was the lowest in the asthmatic patients during the allergic season, when compared to allergic patients after pollen season. Interestingly, mice with miR-155 gene knock-out demonstrated lung remodeling and elevated leukocyte number in BALF and after sensitization, these transgenic mice demonstrated significantly decreased IL-2 and IFNγ expression in comparison to sensitized mice without knock-out, suggesting a protective anti-inflammatory function of miR-155 [39].

MiRNA expression was also analyzed in exhaled breath condensates from asthmatic patients and control subjects, showing decreased expression of several miRNAs in asthma (miR-1248, let-7a, miR-155, miR-21, miR-328, and miR-133a) [23].

Bronchial epithelial brushings from subjects with asthma and healthy controls were analyzed on microarrays, revealing an altered profile of miRNA expression [38,41,54], with the exception of Williams et al., who studied a mild asthma phenotype [55]. Jardim et al. discovered 66 differentially expressed miRNAs on comparing healthy and asthmatic donors [41]. Further validation of their findings was concentrated on miR-203, implicated in immunological disorders. Target predictions have identified *AQP4*, a gene encoding a member of the aquaporin family, as a possible target for miR-203. *AQP4* transcript expression was significantly higher in asthmatic donors.

In a study by Solberg et al. the authors found significant differences in the expression of 217 miRNA genes when comparing steroid-naive asthmatic patients and healthy controls, while 200 miRNA genes differed between steroid-using asthmatics as compared to steroid-naïve patients; 170 of these miRNAs were shared between these two asthmatic groups [54]. Treatment with inhaled corticosteroids (ICS) for steroid-naive subjects has shown to normalize the changes in expression of several miRNAs. Interestingly, the members of the miR-34/449 family were highly repressed in asthma; in vivo experiments have shown that their expression levels are also decreased after IL-13 treatment. MiR-449, whose expression is increased during the proliferation and differentiation of airway ciliated cells, has been confirmed to target *NOTCH1* mRNA. As a result, ciliated cells are reduced in number, whereas the number of mucous cells increases, implying an miR-449 contribution to altered epithelial differentiation observed in asthma [42,54]. MicroRNA profiling by Martinez-Nunez et al. detected 33 up-regulated and 91 down-regulated microRNAs when comparing bronchial epithelium obtained from asthmatics and healthy donors [38]. Several microRNAs (miR-18a, miR-27a, miR-128 and miR-155) were down-regulated in asthmatic bronchial epithelium, and biological pathway analysis suggested that they may affect TGF-β, IL-6, IL-8, and IFN signaling. Further study of gene targets has implicated that the microRNA network may exert its effect through a combination of targets (transcription factors *NFATs, NFKB, CREB1, CEBPB, CEBPA, GR, RELA,* signaling transducers MAPK and mTOR), as opposed to the regulation of only a single target [38].

Another miRNA profiling study found that only miR-19a differentiated severe asthma from mild asthma patients and healthy controls, as no significant differences in expression were found for mild asthmatics [40]. Further experiments confirmed that up-regulation of miR-19a increased proliferation rates by inhibiting TGF-beta receptor 2. Over-expression of this miRNA also resulted in reduced levels of the phosphorylated activated form of *SMAD3*.

Selected miRNAs were also analyzed in nasal biopsies from asthmatic patients and healthy donors, revealing differential expression of 10 miRNAs (miR-18a, miR-126, let-7e, miR-155, miR-224 were down-regulated, while miR-498, miR-197, miR-874, miR-143, miR-886-3p were up-regulated) [25]. Analysis of patients with asthma and allergic rhinitis showed further alterations in expression for six miRNAs: miR-224, miR-498, miR-187, miR-874, miR-143, miR-886-3p as compared to the control subjects.

Another approach to microRNA profiling was used by Bartel et al. who first performed microarrays on mice with induced airway inflammation, followed by target prediction for the top 100 dysregulated miRNA genes, and enabled identification of *Creb1*, confirmed to be regulated by miR-17, miR-144, and miR-22 via luciferase reporter assay [43]. Further validation was performed after allergen challenge in mice and in primary human bronchial epithelial (NHBE) cells after IL-13 treatment, confirming down-regulation of the *CREB1/CRTC* pathway.

Another group of small non-coding RNAs recently found in human CD4+ T cells are piwi-interacting RNAs (piRNAs). One of them, piR-30840, was shown to downregulate IL-4 expression and Th2 cell development, thus inhibiting allergic inflammation and allergic asthma [8]. Interestingly, piR-30840 inhibition with an antisense inhibitor resulted in significant upregulation of IL-4 in Th2 cells, suggesting its potential role as an anti-inflammatory agent.

In addition to miRNAs, long non-coding RNAs also play a crucial role in the immune reactions, including allergic asthma [44]. Microarray study has revealed 21 and 19 differentially expressed lncRNAs in airway smooth muscle cells from patients with non-severe and severe asthma, respectively, as compared to healthy individuals. Moreover, *LINC00472, RP5-1158E12.3* and *FKBP1A-SDCBP2* demonstrated similar expression patterns in patients with non-severe and severe asthma as compared to the control group. Interestingly, *PVT1* was significantly increased in patients with severe asthma and decreased in patients with non-severe asthma. PVT1 was also involved in proliferation and IL-6 release in airway smooth muscle (ASM) from patients with severe asthma. Another study analyzing long ncRNAs found that brain cytoplasmic RNA 1 (*BCYRN1*) was dysregulated in airway smooth muscle (ASM) cells, which are crucial during bronchial remodeling in asthma [45]. Using a rat model of asthma, the authors demonstrated increased expression of *BCYRN1* in sensitized rats as compared to control animals. This increase correlated with increased transient receptor potential channel 1 gene expression (*TRPC1*), responsible for enhanced transmembrane Ca^2+^ transport and ASM cell proliferation in asthmatic patients, contributing to bronchoconstriction and dyspnea. Furthermore, it was found that *BCYRN1* induced *TRPC1* expression [56] and overexpression of *BCYRN1* resulted in increased viability, proliferation, and migration rate of ASM from sensitized animal lungs, whereas *BCYRN1* inhibition significantly attenuated these processes [45]. Another study showed reduced expression of *MEG3*, a lncRNA, in the circulating CD8+ T cells in severe asthma, as well as the differential expression of an additional 18 lncRNAs [33].

All of these findings indicate that non-coding RNAs may be involved in the pathogenesis of asthma, not only in the airways regulating local inflammation and airway remodeling, but also on the periphery, thus mediating systemic inflammation in asthma. They also show potential as future biomarkers of this disease.

## 3. NcRNA Studies in Cystic Fibrosis 

Cystic fibrosis (CF) is a genetic disorder caused by mutations in the *CFTR* gene and is the most common chronic, genetically determined, lethal disease. The gene encodes a transmembrane channel that regulates ion exchange between cells and extracellular space. The mutations in the *CFTR* gene result in aberrant protein translation, folding, or trafficking that lead to aberrant regulation of ion exchange and mucus accumulation that are the cause of recurrent airway inflammation and infections decreasing the lung function and shortening the lifespan [57].

Non-coding RNA studies in CF have primarily focused on their role in regulating *CFTR* function, as well as controlling immune responses in CF airways. Several analyses provided evidence that miRNA genes (miR-101, miR-145, miR-223, miR-384, miR-494, miR-509-3p, and miR-1246) regulate *CFTR* expression, as well as anion transport, particularly in patients with F508del mutation [58,59,60,61,62]. Increased expression of miR-145, miR-223, and miR-494 was found in bronchial brushings from CF patients as compared to non-CF brushings [61,62] and demonstrated the complexity in post-transcriptional regulation of *CFTR*. Megiorni et al. found that increased levels of miR-101 and miR-494 repress *CTFR* expression *in vitro* [59], whereas miR-138 was found to repress *SIN3A*, leading to increased *CFTR* levels [63]. The latter study suggested that miR-138 may improve biosynthesis of *CFTR*-DF508 and restore anion transport to cystic fibrosis airway epithelium.

Interestingly, genetic variants located in the target gene region that binds miRNA might also alter the recognition site for miRNA, affecting the binding to the targeted transcript. This has been observed by Amato et al. who found that the *CFTR* polymorphism (rs10234329) enhances the affinity for miR-509-3p, a potential *CFTR* transcript modulator [64]. Moreover, miR-16 was discovered to restore the deltaF508 *CFTR* protein function by regulating the cAMP-activated chloride conductance and by reducing the IL-8 expression [65].

An example of miRNA polymorphisms influencing their maturation was reported by Endale Ahanda [66]. They found three polymorphisms in miR-99b/let-7e/hsa-miR-125a gene cluster derived from the same precursor that influence maturation of their primary transcripts and deltaF508-*CFTR* mutation can induce an upregulation of miR-99b and miR-125a gene expression, indicating that these miRNAs are important in CF pathogenesis.

Confirmation that ncRNAs may be important in CF severity also may be found in the fact that miR-9 was found to downregulate anoctamin 1 (ANO1) by direct binding to the target sequence in this gene [67]. Anoctamin 1 (ANO1) is a Ca2+ activated Cl- channel in airway epithelium that was found downregulated in CF patients, contributing to disease severity. The use of a microRNA target site blocker for miR-9 increased ANO1 activity and compensated the deficient transmembrane conductance in CF [67].

The inflammatory responses in CF, particularly during pulmonary exacerbations in both innate and adaptive immune responses, may be also controlled by ncRNAs that influence gene expression in inflammatory conditions [68]. Numerous miRNAs were found to be dysregulated in CF lungs (reviewed by [69]). In a study by Oglesby et al. [70], the authors observed decreased miR-17 expression in CF bronchial cells that correlated with enhanced inflammatory cytokine (IL-8) production. Another miRNA that increased expression-stimulated release of inflammatory cytokines in airway epithelial CF cells was miR-155 [71]. The authors discovered high expression of miR-155 in CF airway epithelial cells, both in vitro and in vivo, that subsequently indirectly activated PI3K/Akt signaling and contributed to intrinsic MAPK activation and IL-8 stabilization [71]. In another study, an increased expression of miR-145 was negatively correlated with target gene expression of *SMAD3* in nasal epithelium from CF patients, thus suggesting it may influence regulation of the inflammatory pathway of TGF-β1 and explain the abnormalities in *SMAD3*-mediated TGF-β1 signaling observed in CF [58]. 

A study on miRNA profiling in bronchial brushings from CF patients and healthy controls showed numerous dysregulated miRNA genes (56 down-regulated and 36 up-regulated) [72]. Out of these, miR-126 was further studied and its down-regulation correlated with upregulation of its direct target, *TOM1*, that encodes the protein involved in the endosomal trafficking of ubiquinated proteins. Another miRNA significantly elevated in CF brushings, miR-221, was found to down-regulate ATF6, along with miR-145 and miR-494 [70]. Interleukin-8 mRNA expression was measured in bronchoalveolar lavage fluid and bronchial brushings from CF patients and non-CF controls, showing significantly higher levels in CF [70]. MiR-17 was found to be more effective modulator of IL-8 expression than let-7b, and its over-expression caused decreased production of interleukin-8.

The studies on miRNA profile and their use as potential biomarkers of CF are limited. So far, there has been one study suggesting that the miRNA expression profile may play a role in airway disease in CF [73]. The authors found that decreased miR-31 in the CF airways influences cathepsin S (CTSS) expression in epithelial cells via a transcription factor (IRF-1) responsible for host defense cellular response in CF airway epithelial cells. The authors also observed that treating CF bronchial epithelial cells with a miR-31 mimic decreased IRF-1 protein levels as well as catepsin S expression and secretion, thus reducing lung inflammation [73].

Apart from miRNAs, other types of non-coding RNAs have also been studied in CF. Microarray profiling study revealed more than 1000 differentially expressed lncRNAs between bronchial brushings of CF patients and non-CF controls, including *lncRNA XIST, HOTAIR, MALAT* and *TLR8-AS1* [69]. Additional analysis of protein coding transcripts revealed that lncRNAs and mRNAs are linked, since many are divergently transcribed at the promoters of protein coding genes. The authors also found that Toll-like receptor 8 (TLR8) mRNA was highly expressed in CF samples, whereas the level of its antisense lncRNA transcript TLR8-AS1 was low, suggesting it may play a role in viral exacerbations in CF [69].

Moreover, a recent study found that *CFTR* is regulated transcriptionally by a novel long non-coding RNA (lncRNA), BGas, that originates from intron 11 of *CFTR* gene, but is expressed in the antisense orientation relative to the protein coding sense strand [74]. The authors found that BGas affects *CFTR* transcription via alterations of local chromatin and DNA structure and its suppression restores *CFTR* expression and ion channel function. This may offer a novel therapeutic approach and BGas may be a target for activating expression of *CFTR*. The results of ncRNAs in clinical studies and in vitro model of cystic fibrosis are summarized in Table 2.

Another interesting strategy was reported recently by Zarilli et al. [75] who used peptide nucleic acids (PNAs) to prevent miRNA binding to the 3’UTR sequences of *CFTR* mRNA. The authors synthesized 7- and 13-base-long PNAs with the tetrapeptide Gly-SerP-SerP-Gly at their C-end that was complementary to the sequence recognized by miR-509-3p and blocked its binding to *CFTR*, thus enabling its expression. It seems an interesting approach to gene therapy for cystic fibrosis.

## 4. Role of ncRNAs in Epithelial Function during Repair

The airway epithelium acts as a physical barrier against antigens, microbes and viruses, due to the presence of tight junctions, adherens junctions, desmosomes, gap junctions, and hemidesmosomes [76]. Mucociliary clearance and the production of mucus allow the epithelium to trap foreign particles and transport it out of the respiratory system [77].

The epithelium may be damaged by respiratory viruses or microbes or by inhalation of airborne environmental irritants (tobacco smoke, pollutants) [78]. The injury itself causes an acute inflammatory response, resulting in recruitment of immune cells that release cytokines, chemokines, growth factors, and other factors that promote cell migration [79]. A defect in epithelial barrier function results in greater access by environmental allergens, pathogens, and toxicants, a primary cause underlying asthma pathogenesis [80], whereas in cystic fibrosis, ineffective clearing of pathogens leads to inflammation and pulmonary exacerbation [81]. Chronic inflammation of the airways results in aberrant r, decreasing lung function.

The MiRNA expression profile was altered during epithelial wound repair in an in vitro model of a bronchial epithelial cell line (16HBE14o-) with numerous miRNAs down- or up-regulated at different time points as compared to the pre-injury state [82]. Silencing Drosha and Dicer, enzymes crucial in miRNA biogenesis, significantly delayed epithelial repair in 16HBE14o- cells, thus confirming the important role of miRNAs during this process. Further research has revealed that miR-328 is involved in the airway epithelial repair and might regulate actin pathway [83].

## 5. NcRNAs as Mediators of Epithelial Response to Environmental Stimuli

### 5.1. Bacterial and Viral Respiratory Infections

*Pseudomonas aeruginosa* is the leading cause of chronic infections in cystic fibrosis, and infected bronchial epithelial cells from CF patients showed significant up- or down-regulation of 108 lncRNAs in comparison to the controls [84]. These lncRNAs were mostly responsible for regulation of the CTCF binding site and promoter sequence binding. Analysis of a specific signature in infected CF epithelium revealed 17 lncRNA transcripts; four of these were also differentially expressed in patients versus controls: *MEG9, BLACAT1, RP11-477I4.4* and *RP11-1334A24.5*. One of them, MEG9, down-regulated in infected epithelium, correlated with 251 protein coding genes involved in protein binding, regulation of cellular processes, and extracellular matrix, and were present during lung inflammation.

Among viral infections of the airways, the respiratory syncytial virus (RSV) is common human pathogen leading to severe respiratory illness that frequently require hospitalization in infants. A review by Rossi et al. [85] described several miRNAs that are regulated by RSV infection. Of most interest are miRNAs: let-7f, let-7i, miR-24, miR-26b, miR-27a, miR-221, miR-30b, miR-337, miR-339-5p, miR-453, miR-520a, miR-574, and miR-744. Several of these miRNAs were shown to activate or inhibit NF-kB and STAT signaling and cytokine production pathways. Microarray analysis of nasal mucosa identified several miRNAs with altered expression in acute RSV-positive infants (down-regulated miR-34b, miR-34c, miR-125b, miR-29c, mir125a, miR-429 and miR-27b and up-regulated miR-155, miR-31, miR-203a, miR-16 and let-7d) as compared to healthy infants [86]. The majority of these miRNAs regulate the immune system, suggesting they may be important in the immune response to viral infection.

Another class of small non-coding RNAs induced upon RSV infection in human airway epithelium are RNAs derived from transfer RNAs (tRNAs) and called tRNA-derived RNA fragments (tRFs) [87]. One of them, tRF5-GluCTC, was capable of trans-silencing target genes, suggesting that it controls viral replication by suppression of host defense genes. Moreover, a study by Zhou et al. has identified two novel tRFs that promote RSV replication: tRF5-GlyCCC and tRF5-LysCT [88].

Human metapneumovirus (hMPV) is another common virus that belongs to the same family as RSV (*Paramyxoviridae*) and also causes lower respiratory tract infections in infants and children [89]. hMPV infection of bronchial epithelium resulted in altered profile expression of 174 miRNAs. Functional studies revealed that let-7f inhibits hMPV replication and that the M2-2 hMPV protein blocking mitochondrial antiviral signaling inhibits the expression of miR-16 and miR-30a.

Rhinovirus (RV) infections are the most common cause of early-life respiratory infections and the most important cause of asthma exacerbations later in life [90]. Global microRNA profiles generated with NanoString microarrays performed in nasal airway secretions from RV-positive children identified miR-155 as a potential regulator of RV infection [91].

### 5.2. Pollution

Air pollution is associated with asthma development and exacerbations. MicroRNA expression profiling of human bronchial epithelial cells exposed to diesel exhaust particles showed altered expression of 197 miRNAs [92]. Network analysis of the putative targets of the most altered miRNAs has identified genes involved in inflammatory response and cancer. Bleck et al. discovered that miR-375 upregulates thymic stromal lymphoprotein (TSLP) mRNAs and proteins (which in cytokine-linking innate and Th2-adaptive immune disorders are overexpressed as a result of exposure to environmental pollutants) in human bronchial epithelial cells exposed to diesel exhaust particles and ambient particulate matter [93]. Investigators also confirmed that miR-375 downregulates the aryl hydrocarbon receptor (AhR), leading to activation of NF-κB pathway.

Another particle that alters respiratory immune responses is ozone. MiRNA expression profiling in sputum of subjects exposed to ozone inhalation revealed significantly up-regulated expression of 10 miRNAs: miR-132, miR-143, miR-145, miR-199a-3p, miR-199b-5p, miR-222, miR-223, miR-25, miR-424 and miR-582-5p [94]. Predicted targets of these miRNAs were predominantly genes associated with inflammation and immune response.

### 5.3. Cigarette Smoke

Rats exposed to environmental cigarette smoke (ECS) had significantly altered miRNA expression in the lungs [95]. Targets of the most remarkably down-regulated miRNAs (let-7, miR-10, miR-26, miR-30, miR-34, miR-99, miR-122, miR-123, miR-124, miR-125, miR-140, miR-145, miR-146, miR-191, miR-192, miR-219, miR-222, and miR-223) regulate proliferation, gene expression, stress response, apoptosis, and angiogenesis. Several of these miRNAs were also found to be significantly altered in a study comparing smokers to non-smokers [96]. Interestingly, electronic cigarettes were also found to affect miRNA expression profile in human bronchial epithelial cells: expression of 578 miRNAs was altered upon electronic cigarette liquid exposure, whereas 125 miRNAs were dysregulated after liquid vaporization [97].

The results of above mentioned ncRNAs studies are summarized in Table 3.

## 6. Perspectives

Non-coding RNAs have been extensively studied in many diseases, including respiratory illnesses. Identification of ncRNAs and their role in RNA transcription allows for better understanding about the pathogenesis of respiratory diseases. The development of targeted therapy using inhibitors or synthetic ncRNAs could be a breakthrough in the treatment of these diseases. However, taking into account the complexity of ncRNA regulation, it remains a challenge to use them as biomarkers or therapeutic targets.

## Figures and Tables

**Table 1:** Non-coding RNAs (NcRNAs) in clinical and animal models of asthma.

Class of ncRNA	Regulation	Tissue/Cell Type	Species	Condition/Treatment	Validated Target Gene	Ref.
miR-221	↑	Peripheral blood	Human	Asthma	*Spred-2*	[18]
		Lungs	Mouse	Asthma model		
	↑	Lungs	Mouse	Asthma model	*PTEN*	[19]
		P815 mast cells	Mouse	Asthma model		
	↓	Eosinophils (BALF)	Mouse	Anti-sense miR-221	-	[20]
	↑	BMMCs	Mouse	miR-221 overexpression	*Cdkn1b, Kit, Il2Ra*	[21]
	↑	ASM	Human	Asthma	*p27^kip1^, p21^WAF1^*	[22]
let-7	↓	EBC	Human	Asthma	-	[23]
	↓	BALF	Human	Asthma	-	[24]
	↓	Nasal biopsies	Human	Asthma	-	[25]
miR-21	↑	Serum	Human	Asthma	-	[26]
	↑	Lungs	Mouse	OVA, *Aspergillus fumigatus*, IL-13	*IL-12p35*	[27]
	↓	Lungs	Mouse	miR-21 knock-out	-	[28]
	↑	BECs	Human	Asthma	-	[29]
	↓	EBC	Human	Asthma	-	[23]
miR-126	↑	Lungs	Mouse	OVA sensitization	-	[30]
	↑	BECs	Human	Asthma	-	[29]
	↓	Lungs	Mouse	OVA sensitization (miR-126 inhibitor)	-	[30,31]
	↓	Nasal biopsies	Human	Asthma	-	[25]
miR-146	↑	THP-1 cells	Human	LPS and pro-inflammatory cytokines induction	*IRAK1, TRAF6*	[32]
	↓	CD4+ and CD8+ cells	Human	Asthma	-	[33]
miR-192	↓	Peripheral blood	Human	Asthma	-	[34]
miR-485-3p	↑	Whole lung	Mouse	Asthma model	*Spred-2*	[18,20]
		Peripheral blood	Human	Asthma		
miR-223-3p, miR-629-3p, miR-142-3p, miR-338, miR-145	↑	Sputum	Human	Mild-to-moderate asthma	-	[35,36]
miR-155	↓	Sputum	Human	Asthma	-	[37]
	↓	Bronchial epithelium	Human	Asthma	*IL-6, IL-8*	[38]
	↓	Lungs/BALF	Mouse	miR-155 knock-out	*c-Maf*	[39]
	↓	Nasal biopsies	Human	Asthma	-	[25]
	↓	EBC	Human	Asthma	-	[23]
miR-19a	↑	HBEpC	Human	Asthma	*TGF-βR2*	[40]
miR-200	↓	BALF	Human	Asthma	-	[24]
miR-1248, miR-328, miR-133	↓	EBC	Human	Asthma	-	[23]
miR-203	↓	HBEpC	Human	Asthma		[41]
miR-449	↓	HAECs	Human	AntagomiR-449a/b	*DLL1, NOTCH1*	[42]
miR-18a	↓	BECs	Human	Asthma	-	[38]
	↓	Nasal biopsies	Human	Asthma	-	[25]
miR-27a, miR-128	↓	BECs	Human	Asthma	*SMAD2*	[38]
miR-224	↓	Nasal biopsies	Human	Asthma	-	[25]
miR-498, miR-197, miR-874 miR-143, miR-886-3p	↑	Nasal biopsies	Human	Asthma	-	[25]
miR-17, miR-144	↑	Lungs	Mouse	OVA-sensitization	*Creb1*	[43]
miR-22	↓	Lungs	Mouse	OVA-sensitization	*Creb1*	
piR-30840	↑	CD4+ cells	Human	Asthma	*IL-4*	[8]
*LINC00472, RP5-1158E12.3, FKBP1A-SDCBP2*		ASMCs	Human	Non-severe and severe asthma	-	[44]
*PVT1*	↑	ASM	Human	Upregulated in patients with severe asthma as compared to patients with non-severe asthma	-	
*BCYRN1*	↑	Lungs	Rat	Asthma model	*TRPC1*	[45]

BALF: bronchoalveolar lavage fluid; LPS: lipopolysaccharide; OVA: ovalbumin; BMMCs: bone marrow-derived mast cells; ASM: airway smooth muscle, BECs – bronchial epithelial cells, HAEs – human airway epithelial cells, EBC – exhaled breath condensate, HBEpC – human bronchial epithelial cells.

**Table 2:** NcRNAs in cystic fibrosis.

Class of ncRNA	Regulation	Tissue/Cell Type	Validated Target Genes	Ref.
miR-145, miR-384, miR-494, miR-1246	-	Caco-2 cell line	*CFTR*	[60,62]
miR-101, miR-494	↑ (mimic)	HEK293	*CFTR* repressed	[59]
miR-145, miR-223, miR-494	↑	bronchial brushings	*CFTR*	[61]
miR-509-3p, miR-494	↑	primary airway epithelial cells, Calu3	*CFTR* decreased	[62]
miR-138	↑ (mimic)	primary airway epithelial cells, Calu-3, HEK293T, HeLa, CFBE	*CFTR* increased *SIN3A* repressed,	[63]
miR-16	↑ (mimic)	IB3-1 CF lung epithelial cells, CFPAC-1 cells	*CFTR* increased	[65]
miR-9	↑	CFBE41o-, 16HBE14o-	*ANO1*	[67]
miR-17	↓	bronchial cells	Enhanced IL-8	[70]
miR-155	↑	lung epithelial cells	*SHIP1* reduced	[71]
miR-145	↑	nasal epithelium	*SMAD3*	[58]
miR-126	↓	bronchial brushings	*TOM1*	[72]
miR-221, miR-145, miR-494	↑	CFBE41o-, 16HBE14o-, bronchial brushings	*ATF*	[70]
miR-31	↓	PBECs, CFBE41o-, 16HBE14o-, 9HTEo-, CFTE29o-	*CTSS, IRF-1*	[73]
XIST HOTAIR, MALAT, TLR8-AS1	↑ ↓	bronchial brushings	- *TLR8*	[69]
BGas	↓ (inhibition)	CFPAC cells, 1HAEo-, CFBE41o-, 16HBE14o-	*CFTR* increased	[74]

HEK293 – human embryonic kidney cells, IB3-1 – primary human bronchial epithelial cells from CF patients, CFBEs – cystic fibrosis bronchial epithelial cells, CFPAC – human Caucasian pancreatic adenocarcinoma, HBE – human bronchial epithelial cells, HAE – human airway epithelial cells, CFTE – human tracheal epithelial cells from cystic fibrosis, HTE – human tracheal epithelial cells, PBECs – primary bronchial epithelial cells.

**Table 3:** A summary of ncRNA studies in airway cells exposed to different environmental stimuli.

Class of ncRNA	Regulation	Tissue/Cell Type	Species	Condition/Treatment	Validated Target Genes	Ref.
lcRNAs: *MEG9, BLACAT1,* *RP11-477I4.4, RP11-1334A24.5*	↓	bronchial epithelial cells	humans	CF + *Pseudomonas aeruginosa*	-	[84]
miR-34b, miR-34c, miR-125b, miR-29c, mir125a, miR-429 miR-27b	↑	nasal epithelium brushings	humans	RSV	-	[86]
miR-155, miR-31, miR-203a, miR-16 and let-7d	↓				-	
tRF5-GluCTC	↑	lung carcinoma cell line (A549)	humans	RSV	-	[87]
tRF5-GlyCCC, tRF5-LysCT	↑	lung carcinoma cell line (A549)	humans	RSV	-	[88]
let-7f	↑	lung carcinoma cell line (A549)	humans	hMPV	-	[89]
miR-155	↑	nasal airway secretions	humans	RV	-	[91]
miR-513c, miR-513b, miR-513a-5p, miR-923, miR-494, miR-338-5p,	↑	bronchial epithelial cells	humans	DEP	-	[92]
miR-31-3p, miR-26b, miR-96, miR-27a, miR-135b, miR-374a	↓				-	
miR-375	↑	bronchial epithelial cells	humans	DEP, APM	*TSLP, AhR*	[93]
miR-132, miR-143, miR-145, miR-199a-3p, miR-199b-5p, miR-222, miR-223, miR-25, miR-424 and miR-582-5p	↑	sputum	humans	Ozone	-	[94]
let-7, miR-10, miR-26, miR-30, miR-34, miR-99, miR-122, miR-123, miR-124, miR-125, miR-140, miR-145, miR-146, miR-191, miR-192, miR-219, miR-222, and miR-223	↓	lung biopsies	rats	ECS	-	[95]
hsa-miR-337, hsa-miR-18a-3p, hsa-miR-189, hsa-miR-365, hsa-miR-181d	↑	bronchial epithelial cells	humans	ECS	-	[96]
hsa-miR-10b, hsa-miR-150, hsa-miR-338, hsa-miR-362, hsa-miR-17-3p, hsa-miR-15a, hsa-miR-652, hsa-miR-106b, hsa-miR-19b, hsa-miR-106a, hsa-miR-128a, hsa-miR-30a-3p, hsa-miR-128b, hsa-miR-130a, hsa-miR-500, hsa-miR-363, hsa-miR-199b, hsa-miR-223, hsa-miR-625, hsa-miR-99a, hsa-miR-125b, hsa-miR-146a	↓				-	
hsa-miR-218	↓				*MAFG, NAC-1, ECOP, LASP1*	
miR-126a-3p, miR-126-5p, miR-140-5p, miR129a-5p, miR374a-3p, miR-147b	↑	bronchial epithelial cells	humans	EC	-	[97]

RSV: respiratory syncytial virus; hMPV: human metapneumovirus; RV: rhinovirus, DEP: diesel exhaust particles; APM: ambient particulate matter; ECS: environmental cigarette smoke; CF: cystic fibrosis.
